# First National Bridging Workshop on International Health Regulations 2005 and Performance of Veterinary Services Pathway in Kenya

**DOI:** 10.7189/jogh-14-03045

**Published:** 2024-12-02

**Authors:** Mathew Muturi, Athman Mwatondo, Kazuki Shimizu, Khadija Chepkorir, Kanana Kimonye, Mark Nanyingi, Mario I Alguerno, Osman Dar, Dilys Morgan, Mathew Mutiiria, Serge Nzietchueng, Masika Sophie, Jayne Tusiime, Chadia Wannous, Dan Mogaka, Juliet Nabyonga-Orem, Nollascus Ganda, Guillaume Belot, Stephane de la Rocque, Tieble Traore

**Affiliations:** 1 Zoonotic Disease Unit, Directorate of Veterinary Services, Nairobi, Kenya; 2 Zoonotic Disease Unit, Ministry of Health, Nairobi, Kenya; 3 World Health Organization, Kenya Country Office, Nairobi, Kenya; 4 Food and Agriculture Organization of United Nations, Kenya Country Office, Nairobi, Kenya; 5 World Organisation for Animal Health, Paris, France; 6 United Kingdom Health Security Agency, London, UK; 7 Food and Agriculture Organization of United Nations, Emergency Centre for Transboundary Animal Disease, Eastern and Southern Africa, Nairobi, Kenya; 8 Veterinary Epidemiology and Economics Unit, Directorate of Veterinary Services, Kenya; 9 World Health Organization, Regional Office for Africa, Emergency Hub, Nairobi, Kenya; 10 World Organisation for Animal Health Regional Representation for Africa, Nairobi, Kenya; 11 Centre for Health Professions Education, Faculty of Health Sciences, North-West University, Potchefstroom, South Africa; 12 World Health Organization, Geneva, Switzerland; 13 World Health Organization, Regional Office for Africa, Emergency Hub, Dakar, Senegal

The health of humans and animals is interlinked, and endemic, emerging, and re-emerging zoonotic diseases easily pose risks to both humans and animals. Approximately 60% of widely known infectious diseases and 75% of emerging pathogens are of zoonotic origin [1], and the potential impact of zoonotic diseases on citizens’ livelihoods and socio-economic activities is significant. Furthermore, the continued expansion of interaction in the human-animal-environment interface created opportunities for an increase in emerging and re-emerging infectious diseases. In recent years, multiple epidemics such as avian influenza, Lassa fever, Ebola virus disease, Marburg fever, Rift Valley fever, Crimean-Congo hemorrhagic fever, mpox, and, of course, coronavirus disease 2019, have significantly raised awareness of the importance of a multisectoral approach to preventing and responding to these zoonotic diseases. However, the concrete implementation of multisectoral collaboration between sectors remains often challenging. Practically, there are critical gaps in implementing the One Health approach in multiple areas, such as joint leadership and coordination, health information and epidemiology, including surveillance and risk assessment, and resource mobilisation [2]. Therefore, sustainable multi-sectoral coordination, collaboration, and communication mechanisms are to be defined and implemented in a consensual manner at different levels to address these continuously evolving landscapes [3].

## HISTORY OF NATIONAL BRIDGING WORKSHOP

To fulfill a shared responsibility for collaboration between sectors in public and animal health and for combatting zoonotic diseases, the World Health Organization (WHO), the World Organisation for Animal Health (WOAH) and the United Nations Food and Agriculture Organization actively promote and implement inter-sectoral collaborative approaches among institutions and systems [4]. In particular, WHO and WOAH are the two main international organisations responsible for setting standards and guidelines, and these organisations have historically developed various frameworks, tools, and guidance materials for capacity building in different layers. Under the International Health Regulations (IHR) 2005, a legally binding framework for events that may constitute a public health emergency of international concern, State Parties are required to develop, strengthen, and maintain minimum national core public health capacities to detect, assess, notify, and respond to public health threats. WHO supports State Parties in assessing capacities through the IHR Monitoring and Evaluation Framework [5]. For animal health and zoonosis, the Performance of Veterinary Services (PVS) Pathway developed by WOAH works as a benchmark to objectively evaluate the capacities of a country’s veterinary services, identify their strengths and weaknesses, make recommendations for sustainable improvement and investment, and assess their compliance with WOAH international standards on animal health and welfare [6].

While these approaches provided an opportunity for State Parties to understand strengths and weaknesses in their respective functions and activities, make prioritisation, and promote pathways for improvement, their tools and references are different, sometimes hindering a smooth dialogue across sectors [4]. To overcome challenges, WHO, WOAH, and the World Bank jointly developed an operational framework in 2013 [7], followed by establishing joint approaches for animal and human health [8]. Afterwards, to explore overlapping areas between the two sectors when managing zoonotic events, identify synergies and gaps in coordination, and define opportunities for improved coordination, the National Bridging Workshop methodology has been proposed and conducted in several countries, contributing to implementing the One Health approach [4].

## ONE HEALTH APPROACH IN KENYA AND THE FIRST NATIONAL BRIDGING WORKSHOP

Kenya has been actively implementing the One Health approach [9]. This included the establishment of the Zoonotic Disease Unit (ZDU) in 2012. ZDU is an interministerial platform between the Ministry of Health and the Directorate of Veterinary Services. It serves as the country’s One Health office and secretariat to the Zoonoses Technical Working Group [10]. Through the first strategic plan between 2012–17, the collaboration among multiple sectors to prevent and control zoonoses was strengthened, and the prioritisation of zoonotic diseases and their inclusion in the Integrated Disease Surveillance and Response system was conducted, enhancing preparedness and response [9,10].

Meanwhile, the One Health approach has been promoted through WHO and WOAH’s frameworks. In line with the IHR Monitoring and Evaluation Framework, the joint external evaluation mission was conducted in February and March 2017. In line with the tools of the PVS Pathway, Kenya has demonstrated a strong commitment to the program, having conducted several PVS activities since 2007, the most recent being a second PVS follow-up evaluation in February 2019. Following these momenta, the first National Bridging Workshop on WHO-IHR and WOAH-PVS Pathway in Kenya was conducted on 16–19 November 2021.

**Figure 1:**
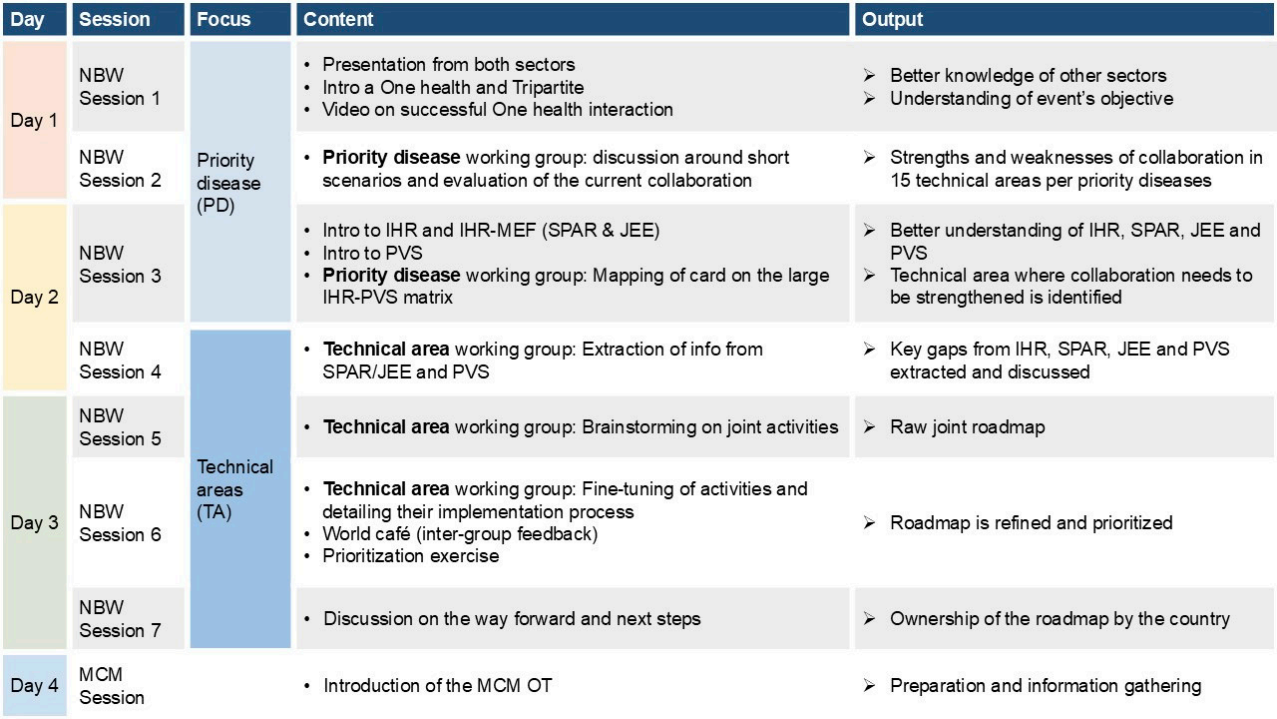
Agenda for National Bridging Workshop in Kenya, November 2021.

The four-day workshop was divided into seven sessions using a facilitator-guided step-by-step approach (**Figure 1**). 65 participants attended the workshop, mainly representatives from the human and animal health sectors and a few wildlife authorities. Around 80% of participants belonged to the national level, while others were from sub-national and district levels. Nearly half of the participants were from public health services, followed by the veterinary and agricultural sectors.

## ASSESSMENT OF COLLABORATION LEVELS AND IHR-PVS BRIDGING

**Figure 2:**
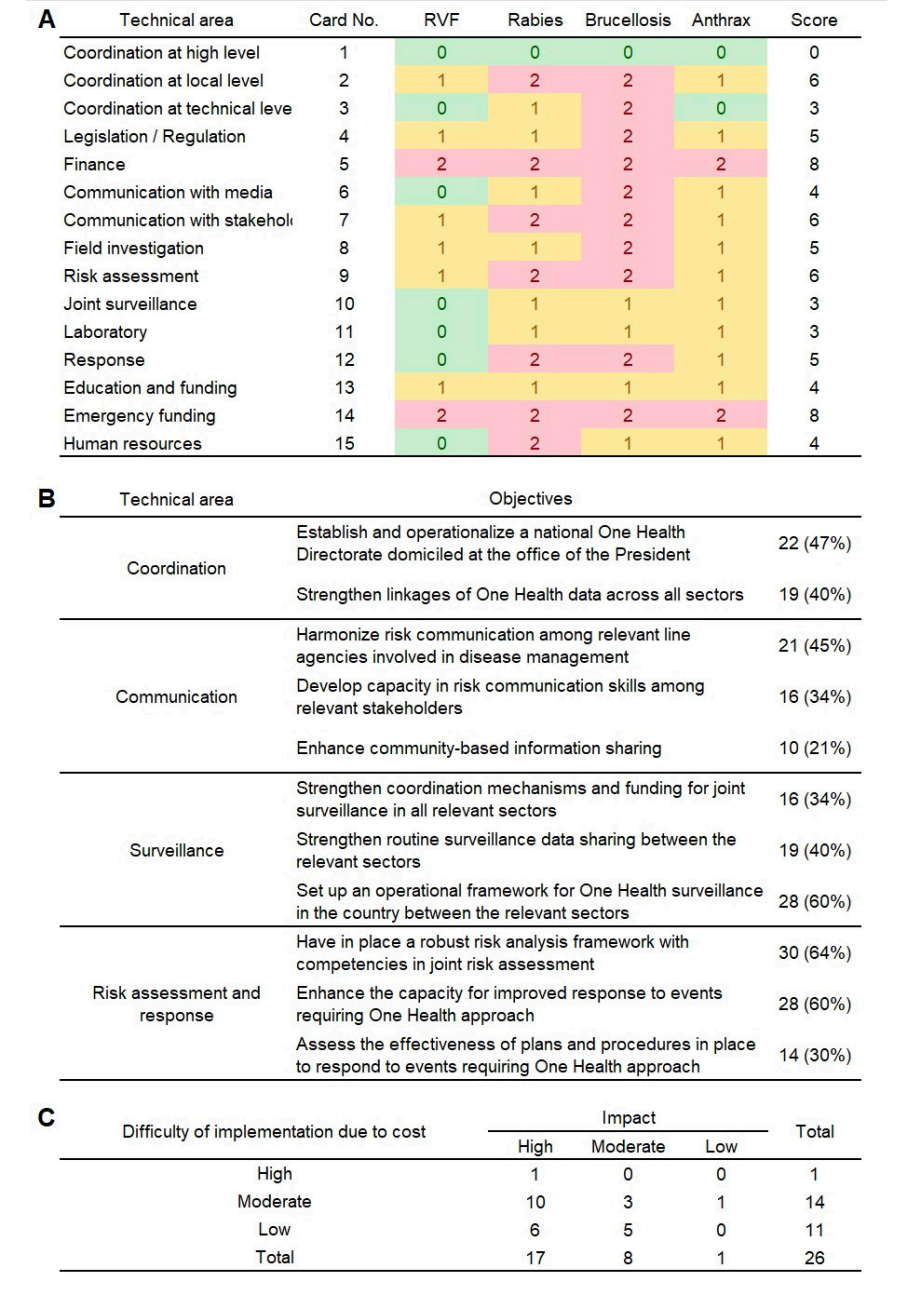
Outputs of National Bridging Workshop in Kenya, November 2021. **Panel A.** Assessment of collaboration levels for 15 key technical areas in Kenya, based on four diseases. For each disease, the performance of collaboration between sectors is color-coded. Green for ‘good collaboration,’ yellow for ‘average collaboration,’ and red for ‘collaboration that urgently needs improvement.’ The score uses a semiquantitative scale: two points for red, one point for yellow, and zero points for green. **Panel B.** Priority objectives of One Health roadmap in Kenya by technical areas. **Panel C.** Number of activities in the One Health roadmap in Kenya, by implementation difficulty and impact.

Four working groups were created to address different scenarios: Rift Valley Fever, Rabies, Brucellosis, and Anthrax. By using diagrammatic arrows to represent the progression of the situation and reflecting on experiences of managing previous outbreaks, each group identified joint activities and areas of collaboration and assessed the level of collaboration between animal and human health sectors in 15 technical areas (**Figure 2**, Panel A).

After presentations of IHR Monitoring and Evaluation Framework, PVS Evaluations and PVS gap analysis, each sector learned about the available tools on the counterpart side, and participants extracted key gaps and recommendations to operationalise the One Health approach. Subsequently, joint areas and activities were mapped onto a giant matrix built with IHR Monitoring and Evaluation Framework and PVS indicators. This enabled participants to identify and understand strengths and weaknesses for further collaboration. Major gaps were openly discussed through a plenary session, and participants agreed to jointly address four priority thematic areas to develop the operational national roadmap and to improve collaboration at the human-animal interface: Coordination, Communication, Surveillance, Risk Assessment and Response.

## JOINT ROADMAP FOR ONE HEALTH

Finally, a planning exercise was conducted to improve inter-sectoral collaboration and develop a concrete and achievable roadmap. Through extracting assessment results and designing the joint roadmap, a total of 26 activities in 11 objectives were raised. Then, the top five objectives of highest priority were selected through an electronic survey, which 47 participants submitted. The most prioritised objective was to ‘have in place a robust risk analysis framework with competencies in joint risk assessment’ in Risk Assessment and Response, which 64% had voted for. This was followed by objectives ‘set up an operational framework for One Health surveillance in the country between the relevant sectors’ in Surveillance and ‘enhance the capacity for improved response to events requiring One Health approach’ in Risk Assessment and Response, both of which were prioritised by 60% of participants. Other priority objectives were summarised (**Figure 2**, Panel B). Activities were classified by cost and impact, and among the 26 activities, 25 (96%) required low to moderate cost for implementation, and 17 (65%) activities were assessed to have a high impact on multisectoral collaboration (**Figure 2**, Panel C). For example, the activity with a high impact but high difficulty of implementation due to cost was ‘coordination of joint One Health activities including zoonotic diseases, food safety, antimicrobial resistance, chemical, and environmental hazards.’ The assessment was due to a complex process, including conducting training for joint rapid response teams at the national and county levels, developing a repository and database for all One Health events, conducting after-action reviews for major One Health events, and participating in cross-border One Health simulation exercises. Detailed activities, dates, costs, impact, responsible institutions, and processes are summarised in the full report.

## WAY FORWARD

The post-workshop survey showed that 100% of participants were fully satisfied or satisfied with the workshop and recommended it to strengthen knowledge about the IHR and PVS Pathway and the collaboration between the human and animal health sectors. The high-level engagement of the ZDU and relevant sectors enabled Kenya to carry out the workshop with a recommended number of participants. Both sectors could agree to take ownership of the results of the 26 key activities and improve collaboration between the relevant sectors. Although further discussions would be necessary on how to incorporate the environmental sector for ZDU [9] and how to mobilise other sectors (eg, legal, finance) and participants from sub-national and district levels, the National Bridging Workshop in Kenya provided an opportunity to respond to a call for cost-effective planning and implementation of One Health programs [11] and achieved the following objectives: 1) increase awareness and understanding of WHO’s role, WOAH’s mandate, the IHR Monitoring and Evaluation Framework and PVS Pathway, the differences and connections, 2) understand the contribution of the Veterinary Services in the implementation of the IHR (2005) and how the results of the PVS Pathway and IHR Monitoring and Evaluation Framework are used to explore strategic planning and capacity-building needs, 3) collectively diagnose and identify strengths and gaps of the collaboration between animal health, wildlife health, environment health and public health services, and 4) identify practical next steps and develop achievable activities for the development and implementation of a joint national roadmap to strengthen collaboration and coordination, strengthened preparedness and control capabilities, and effective, timely prevention.

The roadmap has been successfully endorsed by the government, and it was worth noting that the outcome of the workshop has been linked with several mandated plans, including the National Action Plan for Health Security, to strengthen IHR core capacities. Moreover, the newly developed Tripartite Multisectoral Coordination Mechanisms Operational Tool was introduced to participants [12]. With the prior Tripartite Joint Risk Assessment, the National Bridging Workshop became the beachhead to conduct the pilot of the Multisectoral, One Health Coordination Mechanism Development Workshop and effectively helped launch a new One Health strategic plan in the following year. Furthermore, the establishment of a national One Health technical working group, One Health Rapid Response Teams training and their capacity building, finalisation of the National Emergency Response Operational plan that incorporates One Health multisectoral risk management approach, One Health simulation exercise in the cross-border between Kenya, Ethiopia and Somalia, and the establishment and operationalisation of One Health Units at sub-national levels, could be highlighted as key progresses based on the recommendations from the workshop. The outcome is expected to be used to further enhance and strengthen One Health collaboration and coordination among various Ministries and partners.

The National Bridging Workshop is a dynamic and evolving tool and methodology, which over 50 countries have successfully completed as of June 2024. Each workshop has contributed to the enhancement of this valuable tool, and feedback from participants and organisers in different countries was systematically collected after each workshop and used to refine the methodology, process, and materials. A significant update of the methodology and materials of the National Bridging Workshop is planned, informed by the extensive feedback received from participants across the globe. This continuous improvement process makes it possible to apply accumulated insights and improvements gained from previous iterations and ensures that the National Bridging Workshop remains responsive to the needs of countries and effectively supports the strengthening of One Health collaboration.

The One Health approach represents a transformative opportunity to improve collaboration and resource sharing across human health, veterinary, wildlife, and environmental sectors. By strengthening the collaboration between these sectors, One Health aims to enhance preparedness and ensure more effective prevention, detection, and control of infectious disease outbreaks [8]. The recent evolution of the Tripartite to the Quadripartite, with the inclusion of the United Nations Environment Programme, marks an important milestone in advancing global health security. This expanded collaboration recognises the critical role of environmental factors in the emergence and spread of infectious diseases. Integrating environmental considerations more fully into the One Health framework, the Quadripartite collaboration underscores the need for a holistic response to health threats beyond national and sectoral boundaries. The linkage with the environmental sector is now a key agenda, both nationally and globally, as it addresses the interconnectedness of ecosystems, wildlife, and human health.

Globally, there is a growing commitment to accelerating the One Health approach, with numerous countries and regions adopting it as a core component of their public health strategies. The Quadripartite collaboration facilitates the sharing of best practices, fosters innovation, and supports capacity building, enabling countries to better respond to the evolving challenges posed by infectious diseases and other threats at the human-animal-environment interface. Through this unified effort, the global community is making significant progress towards a more resilient and sustainable system capable of protecting the health and well-being of people, animals, and the environment.
